# Relationships between GABA + and Glx concentrations with age and inhibition in healthy older adults

**DOI:** 10.1007/s00429-025-03017-0

**Published:** 2025-09-27

**Authors:** Ciara Treacy, Sophie C. Andrews, Jacob M. Levenstein

**Affiliations:** Thompson Institute, University of the Sunshine Coast, 12 Innovation Pathway, Birtinya, QLD 4575 Australia

**Keywords:** Magnetic resonance spectroscopy, MRS, Healthy ageing, Inhibition, GABA, Glx

## Abstract

Inhibition represents a core executive function which underlies the ability to suppress interfering or distracting stimuli, thereby building resistance against task-irrelevant information. However, the impact of ageing on inhibitory functioning and the role of neuroplasticity - largely driven by predominant excitatory (glutamatergic) and inhibitory (GABAergic) neurochemicals – remains poorly understood. This study investigated age relationships with neurochemical concentrations (GABA + and Glx) and their associations with inhibitory sub-components in healthy ageing. Participants completed three inhibition tasks (flanker, Stroop, go/no-go), each measuring a different sub-component process, via the PsyToolkit platform. MRS data were acquired in the sensorimotor (SM1; *n* = 71, mean age (SD) = 68.3 (± 9.7) years, 39 females) and prefrontal (PFC; *n* = 58, mean age (SD) = 67.6 (± 9.6) years, 30 females) regions using a HERMES sequence and analysed using OSPREY’s pipeline. After correcting for gender and education, semi-partial correlations revealed no significant relationships between age and GABA + or Glx concentrations in either the SM1 or PFC. Furthermore, after correcting for age, gender and education, partial correlations identified a significant negative relationship between SM1 Glx concentrations and go/no-go error rates, such that greater concentrations of SM1 Glx were associated with greater go/no-go accuracy. The null age-neurochemical results suggest that GABA + and Glx may not uniformly decline during healthy ageing, indicating a more nuanced relationship than previously reported. In addition, our neurochemical-behavioural findings provide neurochemically-and-spatially specific evidence that SM1 Glx concentrations may be important for response inhibition. This result indicates a role for the glutamatergic system in supporting inhibition, independent of age.

**Supplementary Information:**

The online version contains supplementary material available at 10.1007/s00429-025-03017-0.

## Introduction

Successful inhibitory functioning relies upon the ability to voluntarily suppress impulses toward environmental interference and ignore competing distractions, thereby facilitating goal-oriented behaviour (Diamond 2013). Debate persists regarding the impact of ageing on inhibitory functioning, with behavioural evidence supporting an age-related decline (Colcombe et al. 2005; Zhu et al. 2010; Fong et al. 2021; Andrés et al. 2008; Nielson et al. 2002; Kouwenhoven and Machado 2024), and refuting an ageing influence (Hsieh and Fang 2012; Salthouse 2010; Borella et al. 2009; Grandjean and Collette 2011; Fernandez-Duque and Black 2006). Two independent meta-analytical reviews exemplify these contradictory findings and challenge the hypothesis of a general inhibition deficit in ageing, with Verhaegen (2011) failing to identify any specific age-related deficits in inhibition across eight tasks, whilst Rey-Mermet and Gade (2018) detected age-related decrements in only a select number of the eleven evaluated inhibitory tasks. Notably, these meta-analyses identified at least eight different tasks to measure inhibition, highlighting the diverse range of task designs and stimuli employed to evaluate age-related changes. This variability in methodology may be obscuring relationships associated with healthy ageing, exacerbating the inconsistencies observed in the literature.

Cognitive changes observed during normal ageing can, in-part, be explained by neuroplasticity (Burke and Barnes 2006), a process largely driven by the relative balance of predominant excitatory and inhibitory neurochemicals (Duncan et al. 2014). The ratio of excitatory and inhibitory neurons within cortical circuits is under precise, homeostatic control to generate a stable global level of activity (Sohal and Rubenstein 2019). Within the cortex, glutamate (Glu) and γ-aminobutyric acid (GABA), the major excitatory and inhibitory neurochemicals, respectively, are closely related to the excitation-inhibition (E/I) balance (Isaacson Jeffry and Scanziani 2011). Magnetic resonance spectroscopy (MRS) is a non-invasive in-vivo imaging technique to quantify neurochemical concentrations in specific brain regions, with edited experiments capable of isolating low-concentration neurochemicals with substantial signal overlap, such as GABA. This neurochemical is often denoted as “GABA+” due to co-editing of macromolecules at similar resonances (Saleh et al. 2019). MRS quantification of Glu presents a similar challenge due to the significant signal overlap between Glu and glutamine (precursor molecule) resonances, thus, their contributions are often combined and referred to as “Glx” (Ramadan et al. 2013; Mullins et al. 2014). In healthy adults, E/I imbalances have been associated with working memory (Marsman et al. 2017), conflict resolution (de la Vega et al. 2014), perceptual performance (Kondo et al. 2018) and inhibitory functioning (Koizumi et al. 2018). Consequently, the crucial roles of GABA + and Glx in coordinating neural function and stability (Bannai et al. 2020) has prompted MRS investigations to examine their presentation in the ageing brain and role in human behaviour.

While research investigating the impact of healthy ageing on MRS-assessed neurochemical concentrations remains limited, older adults typically exhibit reduced GABA + and Glu (and/or Glx) concentrations across the brain (Porges et al. 2021; Roalf et al. 2020). For example, age-related declines in cortical GABA + and/or Glx concentrations have been reported across the cerebral cortex (Gao et al. 2013; Simmonite et al. 2019; Porges et al. 2017a; Ding et al. 2016) including the motor regions (Grachev and Apkarian 2001; Hermans et al. 2018; Zuppichini et al. 2024; Kaiser et al. 2005; Liu et al. 2024) Furthermore, the relationship between MRS-assessed GABA and Glu (and/or Glx) concentrations and behavioural inhibition in healthy older adults (i.e., ≥ 50 years) remains unclear due to limited evidence. Of these few studies includes work conducted by Hermans and colleagues (2018) who identified that greater SMA GABA + concentrations were associated with greater reactive inhibition, as measured using a stop signal task (SST). Furthermore, recent work by Liu et al. (2024) examined 18 younger (age range = 20–35 years; M_age_ = 22.3 years) and 17 older (age range = 65–85 years; M_age_ = 71.1 years) healthy adults and reported that Glu concentrations negatively correlated with reactive inhibition performance (as measured using the SST) in the younger group, however, this relationship was not observed in the older group. Drawing on additional MRS investigations of inhibition in younger adults, GABA + concentrations have been positively associated with response inhibition (as measured using the go/no-go task) in the striatum (Quetscher et al. 2015), anterior cingulate cortex (Silveri et al. 2013) and superior temporal gyrus (Cheng et al. 2017). Beyond inhibition, GABA and Glu (and/or Glx) concentrations have been cross-sectionally related with other cognitive abilities in healthy older adults, including motor performance (Cassady et al. 2019), sensory discrimination (Puts et al. 2011), general cognitive function (Porges et al. 2017a), fluid processing (Simmonite et al. 2019), working memory (Andersson et al. 2025), and decision making (Rmus et al. 2023). So far, preserving GABA + and Glu (and/or Glx) concentrations in the ageing brain appears to invariably relate to better cognitive outcomes, however further work is needed to confirm these associations across brain regions and tasks in a single study population of healthy older adults. Elucidating the role of GABA+, Glx and their relative balance in inhibitory functioning may help explain the behavioural variability prevailing in ageing.

Prior MRS investigations of inhibition primarily use a singular task to measure response inhibition (e.g., go/no-go or SST), which limits comprehensive assessment of inhibitory functioning, and may obscure behavioural relationships with GABA + and Glx concentrations. An often-overlooked consideration is that behavioural inhibition is not unitary, for example, Rey-Mermet and Gade (2018) propose three sub-components of inhibition: the ability to inhibit (i) distracting information (i.e., flanker), (ii) response interference (i.e., Stroop), and ii) prepotent responding (i.e., go/no-go). In healthy older adults, these inhibitory sub-components appear to be distinct from one-another and show variable age effects (Treacy et al. 2025; Verhaeghen 2011; Salthouse 2010; Fong et al. 2021; Rey-Mermet et al. 2018; Pettigrew and Martin 2014; Kouwenhoven and Machado 2024), therefore, it is probable that they are subserved by distinct mechanisms (Raud et al. 2020) with different neurochemical dependencies. Additionally, in MRS studies important a priori assumptions are made regarding voxel of interest (VOI). Insight from fMRI studies identify the fronto-parietal network as the core system engaged in inhibition, with clusters in the frontal cortex, angular gyrus and SMA (Zhang et al. 2017). Furthermore, inhibiting a prepotent (motor) response also depends upon the fronto-basal ganglia network, including the SM1 (Aron 2011). While prior studies have linked SM1 and PFC GABA+/Glx concentrations to various cognitive domains (Li et al. 2022; Roalf et al. 2020), neurochemical links to inhibitory sub-components have not been investigated in healthy older adults specifically (≥ 50 years).

To address this gap, we clarify the relationships between SM1 and PFC neurochemical concentrations and three sub-components of behavioural inhibition in healthy older adults aged ≥ 50 years. For aim 1, we examined the relationship between healthy ageing, GABA + and Glx concentrations from the SM1 and PFC. We hypothesised that age would be negatively associated with GABA + and Glx concentrations in both brain regions, such that older age would relate to lower neurochemical concentrations. For aim 2, we investigated relationships between SM1 and PFC neurochemical concentrations and behavioural inhibition, as measured using the flanker, Stroop and go/no-go tasks. We hypothesised that GABA + and Glx concentrations would be negatively associated with performance on all three tasks in both brain regions, such that higher neurochemical concentrations would relate to greater inhibitory abilities. As an exploratory aim, we assessed these ageing and behavioural relationships using the E/I ratio, calculated as Glx divided by GABA + concentrations. We hypothesised a positive relationship between ageing and the E/I ratio in both regions, such that older age would relate to a higher E/I ratio. As this ratio is a simple fraction, it can increase due to lower GABA, higher Glx, or differential rates of change in both neurochemicals. To clarify, although we hypothesise both lower GABA + and lower Glx concentrations during ageing in line with prior work (for meta-analyses, see Porges et al. (2021) and Roalf et al. (2020)), the precise trajectory of age-related change is not well understood and may differ between neurochemicals (Thomson et al. 2024). Therefore, within the context of the present study, we conceptualise a higher E/I ratio as reflecting a greater relative imbalance between excitatory and inhibitory neurochemicals. Lastly, we hypothesised that the E/I ratio would be positively associated with performance on all behavioural tasks, such that a higher E/I ratio (i.e., greater neurochemical imbalance) would relate to worse inhibitory abilities.

## Methods

### Participants

This study was approved by the Human Research Ethics Committee of the University of the Sunshine Coast (UniSC; S211620), with data collection occurring at the UniSC Thompson Institute. All participants provided written, informed consent. Healthy older adults aged 50–85 years were recruited from the general community through a study specific webpage, media announcements and local community groups. Eligible participants were right hand dominant, English-speaking, generally healthy individuals absent of any diagnoses pertaining to mild cognitive impairment, psychiatric disorders (e.g., schizophrenia or bipolar), or major neurological conditions (e.g., dementia or Parkinson’s). Additionally, participants were excluded if they presented with MRI contraindications (e.g., pacemaker, stents, metallic foreign bodies or weight exceeding 120 kg), colour-blindness, respiratory conditions (e.g., COPD), cardiovascular conditions (e.g., stroke, TIA or myocardial infarction), prior head injuries (loss of consciousness > 60 min), poorly controlled diabetes, current substance abuse or misuse, or usage of medications known to impact the central nervous system (e.g., antidepressant or antianxiety medication). Eligibility criteria were operationalized via online self-report questionnaires and telephone screening. To determine the required sample size, we referred to prior research also investigating age-relationships with neurochemical concentrations and associations with behaviour (Petitet et al. 2021; Hermans et al. 2018; Gao et al. 2013; Liu et al. 2024). An a priori power analysis was conducted via G*Power software, using the average effect size reported across these studies. For a two-tailed bivariate model with *r* = -0.59, power = 0.80, and α = 0.05, a minimum sample size of *n* = 20 participants was required. In total, 81 individuals satisfied eligibility screening and were enrolled in the study. Of these, two individuals did not complete the MRI due to scanning contraindications, two individuals had incomplete spectroscopy datasets (*n* = 1 incomplete SM1, *n* = 1 incomplete PFC), and one individual was excluded post-hoc due to unreported cognitive difficulties impacting task compliance. Therefore, the MRS cohort consisted of 77 participants. After data quality and accuracy verification, the final MRS sample was reduced to a maximum of 71 participants for the SM1 dataset (M_age_ = 68.2 years, 39f) and 58 participants for the PFC dataset (M_age_ = 67.6 years, 30f), thus, respective ROI sample sizes were adequate to test hypotheses (see Table 1 for univariate statistics).

**Table 1:** Participant sample characteristics

	*n*	Mean(± SD)	Median	Min - Max
SM1 Sample Characteristics				
Age (years)	71	68.28(± 9.67)	70.38	50.56–84.77
Gender (f, m)	39, 32	-	-	-
Education (years)	71	16.36(± 3.79)	16.00	9.00– 26.00
GABA+/tCr	68	0.38(± 0.17)	0.37	0.04–0.80
Glx/tCr	71	1.02(± 0.32)	1.00	0.43–1.83
E/I ratio	68	3.65(± 2.94)	2.50	0.66–14.68
Go/no-go (error rate)	70	9.63(± 8.54)	6.67	0.00–34.33
Go/no-go (bis)	70	2.09(± 1.15)	2.11	-3.47–2.09
Stroop Effect (reaction time)	71	88.95(± 65.99)	73.00	-11.50–290.00
Stroop Effect (error rate)	71	1.48(± 3.30)	0	-3.33–13.43
Flanker Effect (reaction time)	71	22.96(± 30.84)	28.00	-73.00–109.00
Flanker Effect (error rate)	71	0.86(± 2.86)	1.28	-7.37–8.00
PFC Sample Characteristics				
Age (years)	58	67.60(± 9.55)	69.84	50.56–84.77
Gender (f, m)	30, 28	-	-	-
Education (years)	58	16.27(± 3.63)	16.00	9.00–26.00
GABA+/tCr	47	0.33(± 0.16)	0.34	0.04–0.68
Glx/tCr	57	1.32(± 0.34)	1.35	0.53–2.06
E/I ratio	46	5.83(± 5.71)	3.77	0.78–26.86
Go/no-go (error rate)	58	9.50(± 8.11)	6.67	0.00–33.33
Go/no-go (bis)	58	1.90(± 1.02)	2.00	-1.98–1.90
Stroop Effect (reaction time)	58	82.86(± 54.67)	73.75	-11.50–205.50
Stroop Effect (error rate)	58	1.61(± 3.75)	0	-3.33–15.10
Flanker Effect (reaction time)	58	24.86(± 31.31)	32.50	-73.00–109.00
Flanker Effect (error rate)	58	0.41(± 2.72)	0.85	-6.77–5.41

n (sample size), female (f), male(m), sensorimotor (SM1), prefrontal cortex (PFC), relative to total creatine (/tCr), excitation/inhibition (E/I), balanced integrated score (*bis*)

### Behavioural task paradigms

**Fig. 1:**
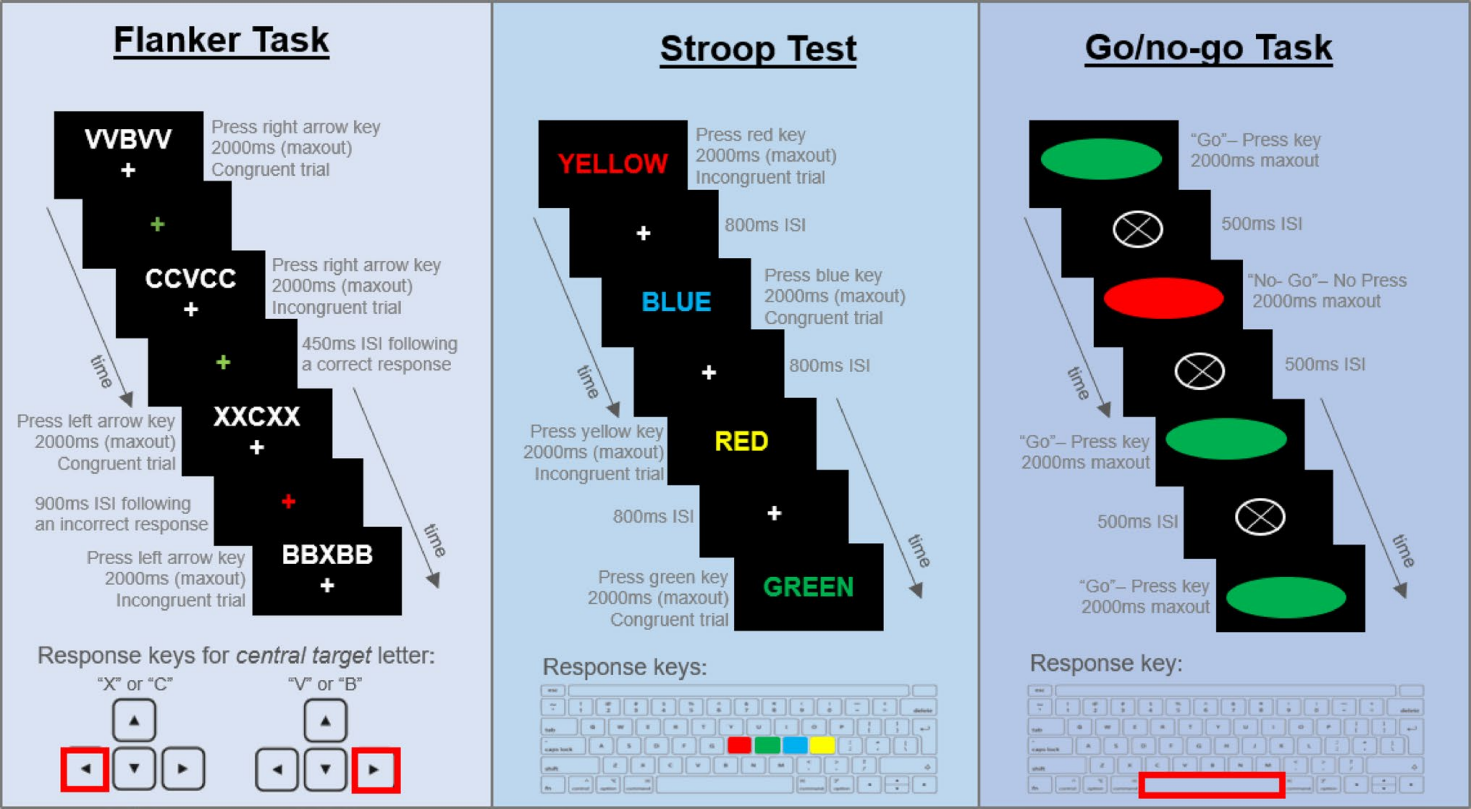
Behavioural task diagrams for the flanker, Stroop and go/no-go inhibitory measures, adapted from Treacy et al. (2025). Inter-stimulus-interval (ISI)

The specifics of the behavioural paradigms used in the present study have been reported elsewhere (Treacy et al. 2025). In short, distractor processing was assessed using a 5-minute letter flanker task (Eriksen and Eriksen 1974), in which participants responded to a central target letter flanked by congruent or incongruent distractors. Letter strings (e.g., “CCXCC”) were presented for 2000ms, and participants were instructed to respond using the left arrow key for “X” or “C” and right arrow key for “V” or “B”. After 30 practice trials participants completed 150 experimental trials, with both blocks incorporating performance feedback (approximately equal proportion of congruent and incongruent trials). Interference control was assessed using a 4-minute Stroop colour and word task (Golden 1978), comprising congruent and incongruent trials. Colour words (“red”, “green”, “blue”, “yellow”) appeared for 2000ms, printed in either a matching (congruent) or mismatched (incongruent) colour. Participants responding to the print colour only, by pressing colour-coded keys marked with corresponding stickers. After 16 practice trials with performance feedback, participants completed 120 experimental trials without feedback (approximately equal proportion of congruent and incongruent trials). Lastly, response inhibition was assessed using a 4-minute go/no-go task, featuring green (“go” stimulus) and red (“no-go” stimulus) ellipses. Each stimulus appeared for 2000ms, and participants were instructed to press the spacebar for the green (“go”) stimuli and withhold a response for the red (“no-go”) stimulus. The task used a 4:1 ratio of “go” to “no-go” trials to establish a habitual response tendency. After 25 practice trials with performance feedback, participants completed 225 experimental trials absent of feedback. These three inhibition tasks were administered via the PsyToolkit platform v3.4.2 (Stoet 2010, 2017), see Fig. 1 for task diagrams. Upon arrival at the UniSC Thompson Institute, participants were seated in a quiet room at arm’s length from a DELL laptop screen (15.6” Precision 5530 model, intel UHD graphics 630), which was used to administer the PsyToolkit behavioural experiments. The aforementioned task timings reflect the “real-test” period, task instructions and practice trials are not included in these time estimations. Participants could take a short break (~ 5 minutes) in between each task before the next set of instructions were given. For each task, participants were directed to maintain finger contact with the response key/s for the duration to ensure accurate record of reaction times and detection of slips of action. Further, participants were also instructed to give equal importance to both the speed and accuracy of their responses. These behavioural experiments were administered within 1-hour of MRI scanning.

We computed standard performance outcome measures related to inhibition and, where appropriate, speed-accuracy trade-off scores. Specifically, performance on the congruence tasks (i.e., flanker and Stroop) was measured by comparing congruent and incongruent trials in terms of median reaction times and error rates, calculating a congruence effect difference score for each. Go/no-go performance was measured using error rate (i.e., proportion of commission errors) and a balanced integrated score (bis; Liesefeld and Janczyk (2019)), which accounts for speed-accuracy trade-offs to maintain “real” effects. The go/no-go bis was calculated using the standardised mean difference between the proportion of accurate responses and reaction times on correct trials (reaction times > 700ms or < 200ms were removed), such that *bis* = z(x̅ percentage correct) - z(x̅ reaction time). As the bis was the only performance measure in this study where higher scores indicate better performance, this measure was reverse scored to facilitate hypothesis testing (i.e., higher scores on all behavioural measures represents poorer performance).

### MRI data acquisition and processing

**Fig. 2:**
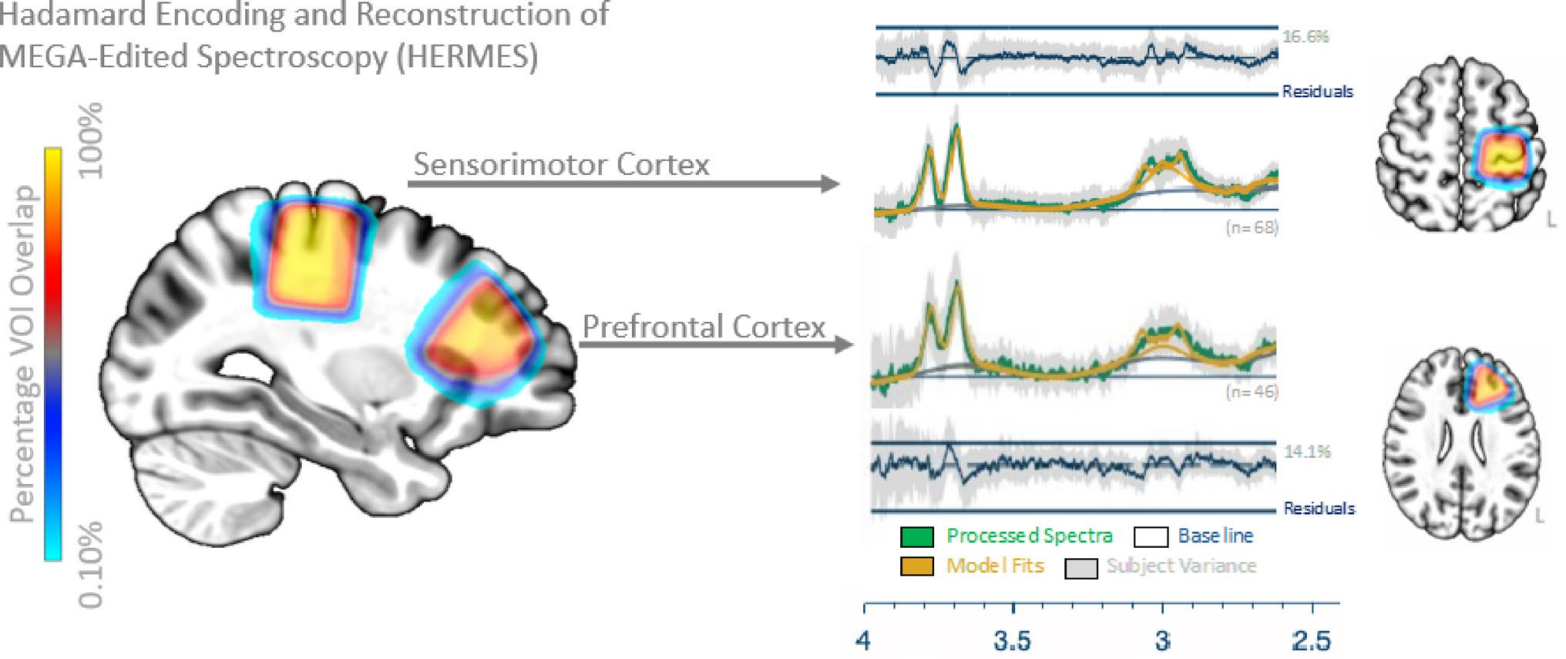
Frequency plot of MRS VOI locations in the left hemisphere (the SM1 and PFC) and corresponding model fits

For all participants, MRI brain scans were performed on a 3T Siemens Skyra (Erlangen German) via a 64-channel head and neck coil. Following localisers, a T1-weighted magnetization-prepared rapid gradient echo (MPRAGE: TR = 2200ms, TE = 1.71ms, TI = 850ms, flip angle = 7°, voxel resolution = 1mm^3^, FOV = 208 × 256 × 256, PAT-GRAPPA = 2, TA = 3:57) was included to plan the MRS voxel placement. Single voxel MRS data were acquired in two different voxels of identical dimension using a Hadamard Encoding and Reconstruction of MEGA-Edited Spectroscopy sequence (HERMES) sequence (Chan et al. 2016), optimized for measuring GABA and GSH (VOI: 3cm^3^, TR = 2000ms, TE = 80ms, flip angle = 90◦, averages = 320, edited pulse 1 = 1.9pmm, edited pulse 2 = 4.56ppm, edited off pulse = 7.5ppm, TA = 10:48). Specifically, voxels were placed in the SM1 region, targeting the “hand region” on the precentral gyrus, and PFC region of the left cortex (Fig. 2). The full MRS post-processing and analysis procedures have been reported elsewhere (Levenstein et al. 2025). In brief, zero T1-weighted scans were removed based on poor image quality or inaccurate VOI placement. MRS data was analysed using OSPREY’s standard processing and fitting pipeline (v.2.4.0, Oeltzschner et al. (2020)), with all MRS data and experiment conditions (i.e., Sum and Diff 1) undergoing visual inspection to verify data quality and accuracy of model fits (Fig. 2). Presented neurochemicals are expressed as a ratio over creatine (Cr) and phosphocreatine (PCr), combined as tCr, and the E/I ratio was calculated by dividing Glx by GABA + concentrations. For the SM1 MRS dataset, the following exclusions were identified: six spectra from the Sum experiments were of poor data quality, impacting the accuracy of model fits for Cr and PCr, resulting in a listwise exclusion for all neurochemicals of interest since tCr was our reference. Furthermore, three Spectra from Diff 1 experiments were of poor data quality impacting the accuracy of model fits for GABA and MM3co (combined as GABA+), resulting in case-wise exclusion. For the PFC MRS dataset, the following exclusions were identified: three spectra from the Sum experiments were of poor data quality, impacting the accuracy of model fits for Cr and PCr, resulting in a listwise exclusion for all neurochemicals of interest. Twenty-eight spectra from Diff 1 experiments were of poor data quality impacting the accuracy of twenty-seven model fits for GABA + and seventeen model fits for Glx, resulting in case-wise exclusion. Thus, the final MRS sample was reduced to a maximum of *n* = 71 participants for the SM1 dataset, and a maximum of *n* = 58 participants for the PFC dataset, see Table 1 for full descriptive summary.

### Statistical analyses

All data statistical analyses were performed using R Statistical Software v4.3.2 (R Core Team 2023). Outliers were identified using z-scores, with z-scores > 3.29 or < 3.29 considered as being extreme cases requiring correction. Outlier adjustment was performed as required using the z-score standard deviation transformation method, whereby extreme cases were adjusted to one unit above or below the nearest value existing within acceptable z-score ranges (Tabachnick et al. 2013). The splithalf package in R (version 0.8.2) was utilised to estimate the internal consistency of inhibition measures, implementing a permutation-based splithalf approach with 5000 random splits (Parsons et al. 2019; Parsons 2021). For the SM1 cohort, one outlier datapoint was adjusted in each of the GABA+, E/I, go/no-go, and flanker datasets, whilst three outlier datapoints were adjusted in the Stroop data. For the PFC cohort, one outlier datapoint was adjusted in each of the Glx, E/I, and flanker datasets, whilst three outlier datapoints were adjusted in the Stroop data. For aim 1, semi-partial correlations (Spearman’s *rho*) were performed to assess the relationship between age and neurochemical concentrations in the SM1 and PFC regions, residual correcting MRS measures for gender and education to isolate age-effects. As only one variable in each comparison was residual-corrected (i.e., age was entered unadjusted), these are referred to as semi-partial correlations. For aim 2, partial correlations (Spearman’s *rho*) were performed to assess the relationships between neurochemical concentrations (SM1 and PFC) and inhibitory performance, residual correcting MRS and behavioural measures for age, gender and education to control for demographic-effects. Since both variables of interest were corrected for shared covariates, these are referred to as partial correlations. For the exploratory aim, we repeated the semi-partial and partial correlations, as previously described, using the E/I ratio (SM1 and PFC) in place of neurochemical concentrations. To correct for multiple comparisons in the primary analyses (aim 1 and 2), we applied a Bonferroni correction resulting in a corrected alpha for age-neurochemical semi-partial correlations of *p* ≤ 0.025 (2 tests per VOI), and neurochemical-behavioural partial correlations of *p* ≤ 0.013 (4 tests per behavioural measure).

## Results

### Behavioural results

Spearman-brown corrected reliability estimates indicated moderate to good reliability for the Stroop and go/no-go tasks, but poor reliability for the flanker task (SB ≤ 30; refer to Online Resource 1). No significant correlations emerged between the three inhibition measures after correcting for multiple comparisons (Refer to Online Resource 2). After residual correcting inhibition measures for gender and education, age was negatively associated with go/no-go bis, reflecting poorer overall go/no-go performance with age (refer to Online Resource 3). Age was also positively associated with Stroop interference effects (reaction time and errors), indicating slower and less accurate performance with age (refer to Online Resource 3).

### Relationships between healthy ageing and neurochemistry

*Primary results:* As shown in Table 2, after residual correcting for gender and education, no significant correlations were observed between age and GABA+/tCr or Glx/tCr in either the SM1 or PFC regions (for figure, refer to Online Resource 4). In addition, we also tested non-linear relationships between age and MRS measures using the nlcor package in R, which also produced non-significant results (refer to Online Resource 5).

To confirm these null findings, we conducted a post-hoc analysis accounting for atrophy, which can influence age-related neurochemical changes (Porges et al. 2017b). This analysis used raw neurochemical concentrations (i.e., not normalised to tCr) and included grey matter and white matter voxel fractions as confounders of no interest, following a residual correction approach (Petitet et al. 2021; Scholl et al. 2017). This correction method thereby controls for atrophy without assuming metabolite distributions across tissue types, remaining agnostic to the differential effects of healthy ageing on brain tissue. Consistent with the primary analysis, this post-hoc approach also produced non-significant results (refer to Online Resource 6). Notably, no correlation was identified between age and tCr (see Table 2). For completeness, correlations between neurochemicals and across brain regions are provided in supplemental material (refer to Online Resource 7) as well as correlations between grey matter voxel fractions, neurochemical concentrations and inhibition measures (refer to Online Resource 8).

**Table 2:** Semi-partial correlations between age and neurochemical concentrations

	Age (years)	
	Coefficient (rho)	*p*-value (uncorrected)
SM1 Region		
GABA+/tCr	-0.002	0.986
Glx/tCr	-0.114	0.342
tCr	0.106	0.379
PFC Region		
GABA+/tCr	-0.021	0.891
Glx/tCr	-0.223	0.095
tCr	0.110	0.410

Neurochemical concentrations were residual corrected for gender and education

*Exploratory results*: As shown in Table 3, after residual correcting for gender and education, no significant correlations were observed between age and E/I ratio in either the SM1 or PFC regions.

**Table 3:** Semi-partial correlations between age and E/I ratio

	Age (years)	
	Coefficient (rho)	*p*-value (uncorrected)
SM1 Region		
E/I ratio	-0.074	0.548
PFC Region		
E/I ratio	-0.116	0.440

Excitation/inhibition (E/I) ratio was residual corrected for gender and education

### Relationships between neurochemistry and behavioural Inhibition

**Fig. 3:**
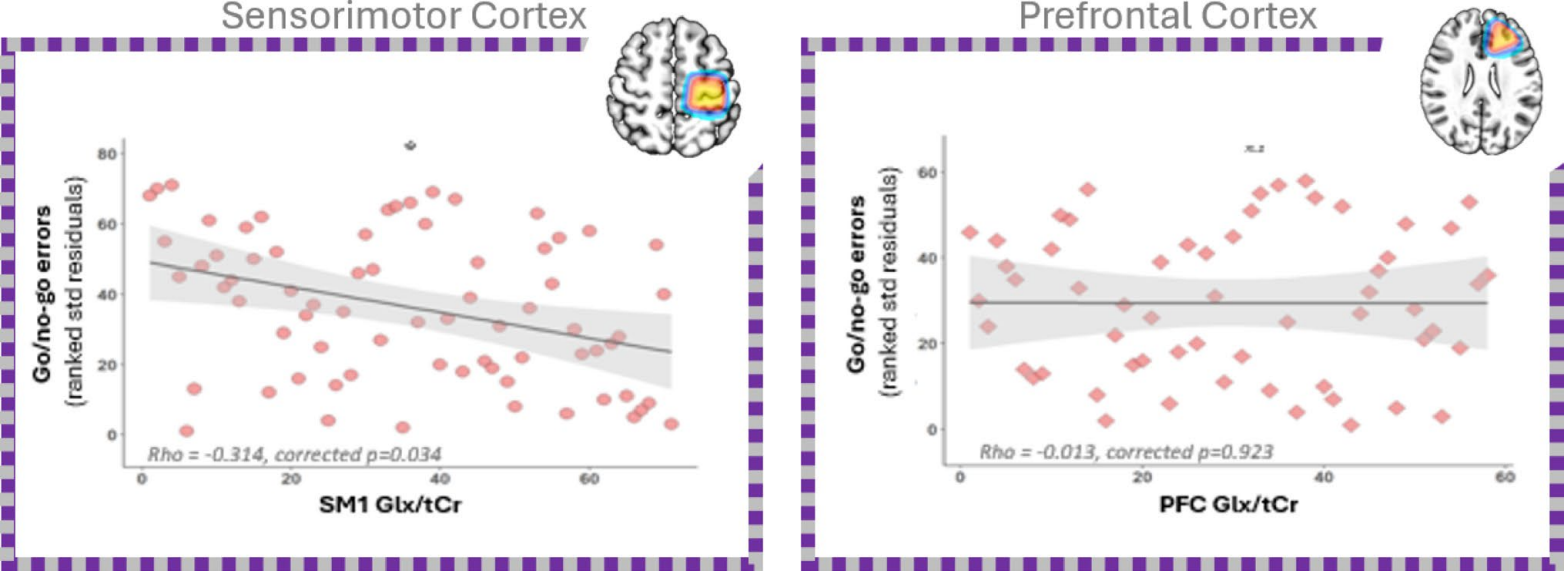
On the left, the primary significant neurochemical-behavioural relationship between SM1 Glx and go/no-go errors. On the right, corresponding non-significant relationship in the PFC region. SM1 data represented by circular points, PFC data depicted using rhombus points. * = *p* < 0.05 corrected; non-significant (n.s)

*SM1 results*: Following multiple comparisons correction, results from partial correlations, residual correcting SM1 neurochemical and behavioural measures for age, gender and education, revealed a significant negative relationship between SM1 Glx/tCr concentrations and go/no-go error rates, such that greater concentrations of Glx/tCr in the SM1 region were associated with greater accuracy on the go/no-go (corrected *p* = 0.034), see Table 4; Fig. 3. SM1 Glx/tCr concentrations were not significantly associated with flanker or Stroop performance (Table 4). After correcting for age, gender, education and multiple comparisons, SM1 GABA+/tCr concentrations were not significantly associated with flanker, Stroop or go/no-go performance (Table 4).

*PFC results*: After correcting for multiple comparisons, age, gender and education, no significant partial correlations were identified between PFC GABA+/tCr or Glx/tCr and inhibitory performance, as indexed using the flanker, Stroop and go/no-go (Table 4).

**Table 4:** Partial correlations between neurochemical concentrations and behavioural Inhibition

	Go/no-go		Flanker Effects		Stroop Effects	
	Error rates	Bis-*r*	Reaction time	Error rates	Reaction time	Error rates
SM1 Region						
GABA+/tCr	0.093	0.038	0.017	0.020	-0.081	-0.140
Glx/tCr	-0.314*	-0.079	-0.048	-0.138	-0.080	-0.022
PFC Region						
GABA+/tCr	-0.183	0.098	-0.397	0.101	0.074	-0.266
Glx/tCr	-0.013	0.142	0.089	-0.253	-0.318	-0.115

Neurochemical concentrations and behavioural measures were residual corrected for age, gender and education. sensorimotor (SM1); prefrontal cortex (PFC); reverse scored balanced integrated score (*bis-r*). * = *p* < 0.05 corrected

*Exploratory E/I results*: As shown in Table 5, after correcting for age, gender and education, results from partial correlations revealed a significant negative relationship between SM1 E/I ratio and go/no-go error rates, such that a greater E/I imbalance in the SM1 region was associated with greater accuracy on the go/no-go (uncorrected *p* = 0.023). Furthermore, a significant positive correlation was identified between PFC E/I ratio and flanker effects (reaction time), such that a greater E/I imbalance in the PFC region was associated with more pronounced flanker effects (uncorrected *p* = 0.034), see Table 5.

**Table 5:** Partial correlations between E/I ratio and behavioural Inhibition

	Go/no-go		Flanker Effects		Stroop Effects	
	Error rates	Bis-*r*	Reaction time	Error rates	Reaction time	Error rates
SM1 Region						
E/I ratio	-0.2777*	-0.0478	-0.1363	-0.1725	0.0804	0.0921
PFC Region						
E/I ratio	0.0984	-0.0625	0.3134*	-0.2213	-0.2847	0.1558

E/I ratio and behavioural measures were residual corrected for age, gender and education. sensorimotor (SM1); prefrontal cortex (PFC); excitation/inhibition (E/I); reverse scored balanced integrated score (*bis-r*). * = *p* < 0.05

## Discussion

This MRS study is the first to investigate relationships between sensorimotor and prefrontal GABA + and Glx concentrations and inhibitory sub-components in a healthy ageing population (aged 50–84 years). Surprisingly, we did not identify any relationships between age and GABA + or Glx concentrations in either the SM1 or PFC regions, whether normalising to tCr or including grey matter and white matter fractions as confounding regressors (Scholl et al. 2017; Petitet et al. 2021). This result is incongruent with previous research in healthy older adults, challenging the notion that these neurochemical concentrations invariably decline as a function of age. For GABA + from SM1 and PFC, no significant relationships were identified with any of the inhibitory sub-component’s performance metrics. Importantly, we identified that Glx concentrations in the sensorimotor cortex are significantly associated with a distinct sub-component process of behavioural inhibition, namely response inhibition (as measured using the go/no-go task). This MRS finding provides regionally specific evidence that individual differences in SM1 glutamatergic function, rather than age per se, may underlie variability in response inhibition in older adults.

### No relationship between age and GABA + or Glx concentrations

In a sample of healthy older adults aged 50–84 years, we did not identify a relationship between age and GABA + or Glx concentrations in either the SM1 or PFC regions. These results were unexpected, contradicting our first hypothesis. Prior MRS studies have reported an age-related decline in both GABA + and Glx concentrations across the brain (Porges et al. 2021; Roalf et al. 2020), including motor (Hermans et al. 2018; Kaiser et al. 2005) and frontal (Rmus et al. 2023; Porges et al. 2017a) regions. However, other regions such as the IFG and hippocampus have shown no age-related differences in GABA + and Glx concentrations (Andersson et al. 2025). It is important to point out that few MRS-ageing studies have been conducted in healthy older adults (aged ≥ 50 years). Of these limited studies includes cross-sectional work conducted by Porges and colleagues (2017a) who assessed 94 healthy older adults (mean age = 73.1 (± 9.9) years) and reported reduced GABA + concentrations (CSF-correction) in the frontal and posterior (i.e., superior to the splenium and aligned with corpus callosum) brain regions as a function of age. Interestingly, a separate analysis of this data processed with a different tissue correction method did not identify a significant relationship between age and frontal GABA + concentration in the healthy older adult sample. Although various MRS correction method possess certain advantages and disadvantages, with no gold standard, this result highlights that the chosen strategy can impact and/or complicate the interpretation of MRS findings (Porges et al. 2017b), potentially masking or bolstering age relationships. That said, we did not identify an age-relationship with GABA + or Glx when conducting two different correction methods (i.e., referencing neurochemical concentrations to tCr or residual correcting for grey matter and white matter tissue fractions) adding weight to the current results. Furthermore, a recent longitudinal study conducted by Zuppichini et al. (2024) in 30 healthy older adults identified that as age increases SM1/ventrovisual GABA + concentrations decrease. Interestingly, no significant cross-sectional relationships were identified between age and GABA + in this cohort, suggesting that assessing within-person age-related neurochemical changes may be more sensitive than group-wide analyses. This reveals another methodological consideration which may provide some explanation for our null age-neurochemical results. Moreover, conflicting results have been reported in younger populations, for example, Mikkelsen et al. (2017) and Aufhaus et al. (2013) did not identify significant age-related changes in GABA + concentrations in healthy adults aged 18–48 years and 21–53 years, respectively. Altogether, these results highlight that the influence of age on GABA + is not clear-cut. Additionally, several studies assessing age-related GABA+/Glx changes do so by statistically comparing separate groups of younger and older adults (Simmonite et al. 2019; Grachev and Apkarian 2001; Hermans et al. 2018; Kaiser et al. 2005; Liu et al. 2024; Rmus et al. 2023). Although this categorical approach elucidates relative, between-group differences, these comparisons do little to characterise ageing trajectories. Indeed, some prior MRS studies have reported age-related declines in GABA+ (age range = 20–76 years; Gao et al. (2013)) and Glx (age-range = 20–70 years; Ding et al. (2016)) with continuity across the lifespan, but a notable gap persists in our understanding of the neurochemical changes occurring in the older adult brain. By placing our results in the context of the existing literature, the seemingly inconsistent ageing results reported here may be influenced by our cross-sectional assessment of healthy older adults specifically (aged ≥ 50 years) and chosen metabolite normalisation method (i.e., referencing to tCr or residual correcting for voxel tissue fractions). It’s crucial to reiterate that evidence delineating MRS-assessed neurochemical trajectories in medically/cognitively healthy older adults remains particularly limited. As such, additional cross-sectional and longitudinal multi-regional MRS research in older adult populations, as opposed to between-group assessments, is required to test the prevailing hypothesis of an age-related decline in GABA + and Glx concentrations. Our null results cast uncertainty on this hypothesis, suggesting that the relationship between older age and neurochemistry may be more nuanced than previously hypothesised.

### Glx, but not GABA+, relates with inhibitory sub-components

In the SM1 region, we identified a negative correlation between Glx and go/no-go performance (error rates), indicating that higher SM1 Glx concentrations benefit the ability to inhibit prepotent responding in healthy older adults. This finding extends on recent work by Liu et al. (2024) who reported that higher Glu concentrations correlated with better inhibitory performance (as measured using the SST) in younger adults (*n* = 18; age range = 20–35 years). This MRS result also complements previous fMRI work that demonstrates the SM1 region’s involvement in the suppression of dominant (motor) responding (Aron 2011), and now suggests that individual differences in SM1 neurochemistry may contribute to response inhibition in a manner that does not appear to be moderated by chronological age. The neurochemical specificity of these findings lends support to the notion that SM1 Glx is indeed playing a functional role. Although there appears to be a mechanistic focus on GABA+ (Li et al. 2022), our results highlight a relationship between Glx and response inhibition, despite the absence of a clear mechanism for this neurochemical (Porges et al. 2021). One explanation relates to glutamate’s role in maintaining cortical excitability and modulating synaptic plasticity (Zhou and Danbolt 2014; Puranik and Song 2024). Individual differences in SM1 Glx may therefore influence the efficiency with which motor-regions interact with broader control networks, including the lateral PFC regions involved in top-down modulation (Aron et al. 2014). Indeed, increased excitatory tone appears to impact the execution of motor task components (Rodríguez-Nieto et al. 2025). However, it’s also possible that higher Glx concentrations may be indicative of a healthier neurochemical system more generally speaking. As such, this significant Glx-response inhibition finding could also reflect differences in neuronal integrity. To clarify this alternate explanation, future studies should further examine the impact of atrophy in the investigated VOI’s and/or consider implementing modalities that capture glutamatergic activity via other neurophysiology measures (e.g., paired-pulse transcranial magnetic stimulation) which could help clarify the role of neuronal integrity. Moreover, additional longitudinal work is needed to clarify the role of neuronal integrity and fully elucidate a causal mechanism. In contrast, no SM1 Glx relationships were identified with the Stroop performance, reinforcing the importance of considering the non-unitary nature of inhibition in ageing research. Indeed, collapsing across inhibitory sub-components may inadvertently obscure meaningful neurochemical relationships. However, due to the low reliability of flanker effects observed in the present work (SB ≤ 30), no firm conclusions can be drawn in relation to this task. This likely reflects several methodological factors. For instance, unstandardised viewing conditions may have altered the visual angle of stimuli (Lee and Pitt 2024), and the task’s relatively short duration may have increased intra-individual variability (Hedge et al. 2018). Moreover, the flanker effect itself likely reflects within-subject phenomena, offering limited between-subject variance, which further constrains its reliability (Hedge et al. 2018; Rouder and Haaf 2019). Taken together, these factors help contextualise the low reliability observed in our sample and underscore the need for caution when interpreting flanker-related findings.

Overall, response inhibition appears to depend on the neurochemistry of the sensorimotor cortex, having distinct Glutamatergic dependencies. With respect to SM1 GABA + concentrations, we did not identify significant relationships with behavioural inhibition measures across tasks. Previous work by Hermans et al. (2018) indicates a role for pre-SMA GABA + in inhibitory functioning in older adults (*n* = 29, mean age = 67.5 (± 3.9) years) using an SST paradigm, however, although the SST and go/no-go are often assumed to measure the same form of inhibition (response inhibition), these inhibition tasks seem to rely on distinct neural mechanisms (Raud et al. 2020) which doesn’t support their interchangeable use. Similarly, using a working memory paradigm, Andersson et al. (2025) found that IFG GABA + concentrations were associated with reduced proactive interference in healthy older adults (*n* = 31, mean age = 73.9 years). These behavioural differences may explain why we did not identify a relationship between GABA + and behavioural inhibition, in conjunction with our different motor ROI and two-fold larger sample size. Although we used a HERMES MRS sequence to improve the specificity of GABA estimates, it is important to acknowledge that these values still include co-edited macromolecular contributions and reflect total GABA + concentrations. Furthermore, MRS based GABA measurements cannot distinguish between synaptic and metabolic pools (Stokes et al. 2014), representing another consideration for interpreting these results.

In the PFC region, no neurochemical-behaviour associations remained significant following Bonferroni corrections for multiple comparisons. Notably, our PFC sample size was considerably smaller than the SM1 because of additional QC and model-fit exclusions (31% reduced for GABA+, 20% reduced for Glx), thus, our PFC analyses were less powered than the SM1 which may explain why these PFC relationships did not surpass corrected statistical thresholds. Altogether, these findings attest that SM1 Glx serves a spatially distinct role in the maintenance of inhibitory functioning, underlining the necessity of considering multiple brain regions to enhance our depth of understanding.

### The E/I ratio

In the present work, we did not detect a relationship between age and the E/I ratio in a sample of healthy older adults. Furthermore, in the SM1 analyses we identified a negative correlation between E/I ratio and go/no-go performance (error rates), suggesting that a higher E/I ratio (i.e., greater neurochemical imbalance) was associated with greater response inhibition. Intriguingly, this association was not observed in the PFC region. This distinction may reflect regionally specific E/I neural dynamics, wherein the computational and functional implications of E/I ratio differ across cortical areas. Furthermore, building on the view that inhibition is a multifaceted construct, E/I ratios in the SM1 appear to differentially influence distinct forms of inhibitory control, namely response inhibition but not interference control. Conceptualised in light of the current findings, higher E/I ratios, representing greater neurochemical imbalance, in the SM1 may enhance sensorimotor responsiveness and facilitate response inhibition. That said, truly understanding what the directionality of the E/I ratio indicates is subject to interpretation, as higher E/I scores can occur from decreasing GABA + concentrations and/or increases in the concentration of Glx, or the scores can reflect GABA + and Glx declining or increasing together at different rates. The GABAergic system has been proposed as primary driver of the E/I ratio (Bi et al. 2020). We identified the un-hypothesised E/I results in the SM1 (higher E/I ratio correlates with better go/no-go performance) where we also detected the strong relationship detected between Glx and go/no-go accuracy. Therefore, if GABA + was indeed driving the E/I ratio, this may have obscured the SM1 associations since GABA + did not relate with behavioural inhibition in this region, perhaps potentiating the unexpected directionality of this relationship. Although this is plausible, elucidating the exact source of these differential reports is challenging as we are unable to conclusively arbitrate between the possibilities driving E/I ratios. Overall, the E/I ratio itself represents quite a simplistic measure. The evidence presented here underscores this ambiguity, which warrants consideration in future research, whilst also highlighting the importance of reporting on the relationships with the neurochemical constituents of the E/I ratio to ensure well-informed conclusions are drawn. It is worth noting that a significant positive correlation was also found between PFC E/I ratio and flanker performance (reaction time); however, given the flanker task’s low reliability (SB ≤ 0.3), this finding should be interpreted with limited confidence.

### Strengths and limitations

By quantifying GABA + and Glx concentrations in two separate brain regions (SM1 and PFC) and targeting three inhibitory sub-components, we were able to demonstrate a spatially specific relationship between neurochemistry and inhibitory functioning in the context of healthy ageing. However, we do acknowledge several potential limitations. This study was cross-sectional in design which restricts the ability investigate temporal relationships and intraindividual neurochemical changes. The included sample were also particularly healthy, highly functioning and educated, which might not be truly representative of the general older adult population. Additionally, the low reliability of the flanker task constrained our ability to draw firm conclusions regarding the generalisability of findings across the three inhibition sub-components. Although the VOI locations of SM1 and PFC were specifically chosen with respect to the inhibitory tasks, quantifying neurochemical concentrations in other VOIs could have further clarify the spatial specificity of these results. Moreover, previous studies have used control sites to assess whether the observed relationships is specific to task-relevant regions or more widespread (Sumner et al. 2010). Future studies employing region-specific control comparisons will further clarify the functional relevance and spatial specificity of observed effects. Additionally, our questions were restricted to the study of GABA+, Glx and E/I ratio, thus, testing these relationships with other neurochemicals would denote the generalisability of these findings and further characterise healthy brain ageing from a neurochemical perspective. Further, we excluded a considerable percentage of the PFC MRS data based on poor data quality (PFC exclusions: 39% of GABA + and 26% of Glx data). With respect to the VOI placement in left PFC using a large voxel size (3 cm^3^), poor data quality was primarily driven by lipid contamination and poor signal/shimming in this frontal region. Although 3 cm^3^ voxel dimensions are advised for the HERMES sequence implemented here, we acknowledge that both the large voxel size and the left PFC placement as potential limitations of the current study. Lastly, suitable unsuppressed water reference data were not acquired for all participants, precluding the use of water-referenced metabolite quantification and certain correction methods (e.g., alpha correction; Porges et al. (2017b)). This is a methodological limitation.

## Conclusion

In the present study, we clarified relationships between SM1 and PFC GABA + and Glx concentrations (as well as their relative balance) and sub-component processes of behavioural inhibition in healthy older adults aged 50–84 years. Contrary to our hypothesis, age was not associated GABA + or Glx concentrations in either the SM1 or PFC regions, nor was age related to the E/I ratio. This unexpected null result casts uncertainty on the prevailing assumption that GABA + and Glx concentrations vary with age among health older adults. Furthermore, we identified that SM1 Glx concentrations are associated with an individuals’ capacity to inhibit prepotent responding, suggesting that response inhibition may rely on sensorimotor Glutamatergic neurochemistry independently of age. Our utility of the E/I ratio as a measure of neurochemical balance produced differential evidence, with the results from one VOI seemingly opposing the other. While this may provide specific insight into neural dynamics, since arbitrating between whether GABA + or Glx is driving E/I ratios remains largely inconclusive, utilising this relatively crude measure of neurochemistry warrants careful consideration in future MRS work. Altogether, the present cross-sectional findings suggest a potential role for SM1 neurochemistry in behavioural function, whereby MRS-assessed Glx concentrations may serve as an important indicator of response inhibition.

## Supplementary Information

Below is the link to the electronic supplementary material.

Supplementary Material 1

## Data Availability

Data analysis/experiment code and materials are openly available at the project’s Open Science Framework page (osf.io/pbr9f). Data sharing has not yet been approved by the UniSC Human Research Ethics Committee, but the data can be made available upon reasonable request by contacting the corresponding author.
