# Supplementary material for: Relationships between GABA + and Glx concentrations with age and inhibition in healthy older adults
Source: Brain Struct Funct. 2025 Sep 27;230(8):149. doi: 10.1007/s00429-025-03017-0 (PMC12476448; doi:10.1007/s00429-025-03017-0)
Supplement: Supplementary file 1 — Supplementary Material 1 [file 429_2025_3017_MOESM1_ESM.docx]

**Supplemental Material**

Title: Relationships between GABA+ and Glx concentrations with age and inhibition in healthy older adults.

Journal: Brain Structure and Function

Authors: Ciara Treacy, Sophie C. Andrews and Jacob M. Levenstein

Affiliation: Thompson Institute, University of the Sunshine Coast, Birtinya, QLD, Australia

Correspondence concerning this article should be addressed to Dr Ciara Treacy ([ctreacy@usc.edu.au](mailto:ctreacy@usc.edu.au))

Online Resource 1: Spearman-Brown corrected reliability estimates for inhibition measures

|  | *SB reliability estimate* |
| --- | --- |
| Flanker Effect (error rate) | *0.30* |
| Flanker Effect (reaction time) | *-0.14* |
| Stroop Effect (error rate) | 0.80 |
| Stroop Effect (reaction time) | 0.78 |
| Go/no-go (error rate) | 0.76 |
| Go/no-go (reaction time) | 0.63 |

Spearman-Brown (SB); Balanced integrated score (bis).

Online Resource 2: bivariate correlations between inhibition measures

|  | Flanker Effect (error rate) | Flanker Effect (reaction time) | Stroop Effect (error rate) | Stroop Effect (reaction time) | Go/no-go (error rate) | Go/no-go (bis) |
| --- | --- | --- | --- | --- | --- | --- |
| Flanker Effect (error rate) | - | - |  |  |  |  |
| Flanker Effect (reaction time) | - | - |  |  |  |  |
| Stroop Effect (error rate) | *rho* = -0.042 *p_raw_* = 0.710  *p_co_*_r_ = 1.000 | *rho* = 0.160 *p_raw_* = 0.158  *p_co_*_r_ = 1.000 | - | - |  |  |
| Stroop Effect (reaction time) | *rho* = -0.020 *p_raw_* = 0.857  *p_co_*_r_ = 1.000 | *rho* = 0.005 *p_raw_* = 0.966  *p_co_*_r_ = 1.000 | - | - |  |  |
| Go/no-go  (error rate) | *rho* = -0.057 *p_raw_* = 0.617  *p_co_*_r_ = 1.000 | *rho* = -0.102 *p_raw_* = 0.370  *p_co_*_r_ = 1.000 | *rho =* 0.042 *p_raw_ =* 0.714  *p_cor_ =* 1.000 | *rho* = 0.187 *p_raw_* = 0.099  *p_co_*_r_ = 0.790 | - | - |
| Go/no-go  (bis) | *rho* = 0.177 *p_raw_* = 0.118  *p_co_*_r_= 0.946 | *rho* = 0.051 *p_raw_* = 0.654  *p_co_*_r_ = 1.000 | *rho =* -0.282 *p_raw_ =* 0.012  *p_cor_ =* 0.095 | *rho* = 0.230 *p_raw_* = 0.041  *p_co_*_r_ = 0.329 | - | - |

Balanced integrated score (bis). praw = uncorrected p value, pcor = corrected p value.

Online Resource 3: Semi-partial correlations between age and inhibition measures

|  | **Age (years)** | |
| --- | --- | --- |
|  | *Coefficient (rho)* | *P-value (uncorrected)* |
| Flanker Effect (error rate) | -0.030 | 0.792 |
| Flanker Effect (reaction time) | -0.051 | 0.654 |
| Stroop Effect (error rate) | 0.337 | **0.002** |
| Stroop Effect (reaction time) | 0.313 | **0.005** |
| Go/no-go (error rate) | 0.045 | 0.696 |
| Go/no-go (bis) | -0.471 | **<0.001** |

N=80 for flanker and Stroop, N=79 for go/no-go correlations. Inhibition measures were residual corrected for gender and education. balanced integrated score (bis).


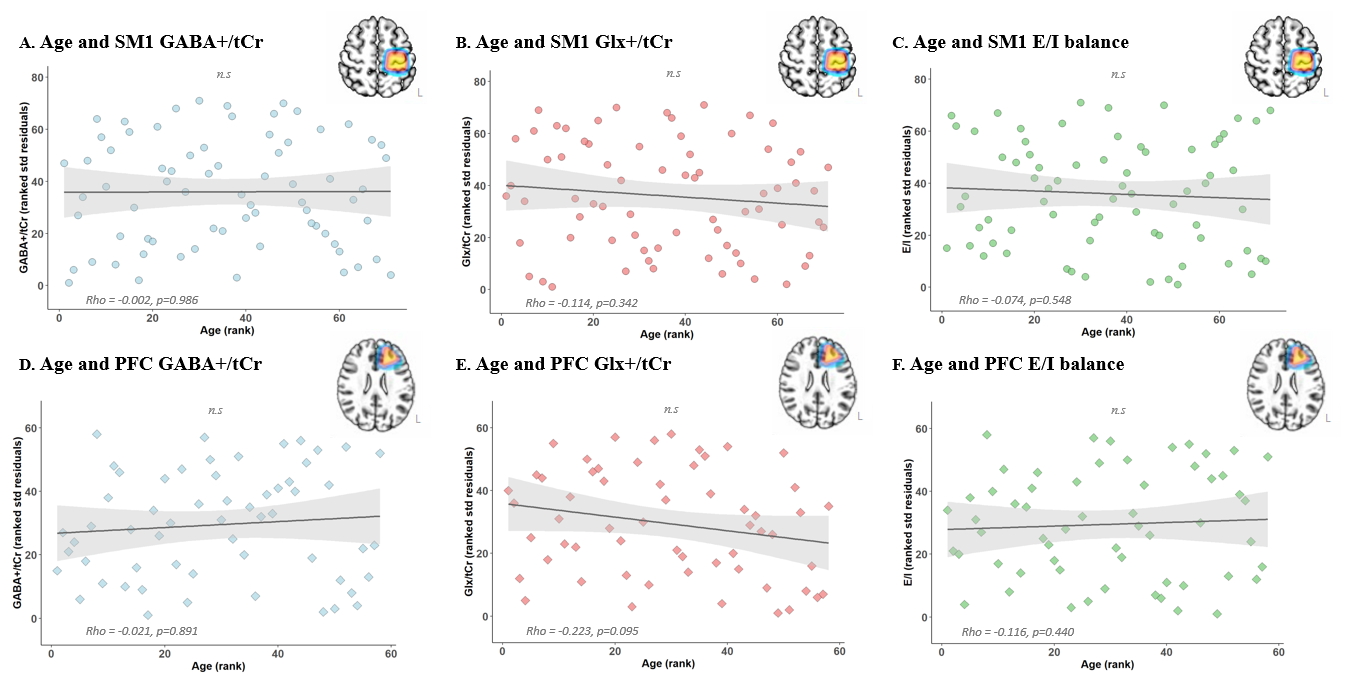
Online Resource 4: Semi-partial correlations (Spearman’s rank) of age, neurochemical concentrations and E/I ratio in SM1 (circle data points) and PFC (rhombus data points) regions. Neurochemical concentrations and E/I ratio are presented as standardised residuals, corrected for gender and education. GABA+, Glx and E/I data are displayed in blue, pink and green, respectively. On the top, age relationships with SM1 GABA+/tCr concentrations (A), SM1 Glx/tCr concentrations (B), and SM1 E/I ratio (C). On the bottom, age relationships with PFC GABA+/tCr concentrations (D), PFC Glx/tCr concentrations (E), and PFC E/I ratio (F). The grey shaded area indicates the 95% confidence interval of the linear regression line. Correlation coefficient (r); uncorrected p-value (p); non-significant (n.s).

Online Resource 5: Non-linear correlations between age and neurochemical concentrations.

|  | **Age (years)** | |
| --- | --- | --- |
|  | *estimate* | *p-value* |
| ***SM1 Region*** |  |  |
| GABA+/tCr | 0.323 | 0.692 |
| Glx/tCr | 0.098 | 0.420 |
| ***PFC Region*** |  |  |
| GABA+/tCr | 0.576 | 0.816 |
| Glx/tCr | 0.210 | 0.120 |

neurochemical concentrations were residual corrected for gender and education.

Online Resource 6: Semi-partial correlations between age and neurochemical concentrations (residual corrected for voxel tissue fractions)

|  | Age (years) | |
| --- | --- | --- |
|  | Coefficient (rho) | p-value (uncorrected) |
| SM1 Region |  |  |
| GABA+ | -0.024 | 0.849 |
| Glx | 0.050 | 0.677 |
| PFC Region |  |  |
| GABA+ | -0.003 | 0.985 |
| Glx | -0.040 | 0.765 |

neurochemical concentrations were residual corrected for grey matter, white matter, gender and education

Online Resource 7: Bivariate correlations between neurochemical concentrations and across brain regions

|  | PFC GABA+/tCr | SM1 GABA+/tCr | PFC Glx/tCr | SM1 Glx/tCr |
| --- | --- | --- | --- | --- |
| PFC GABA+/tCr | - |  |  |  |
| SM1 GABA+/tCr | *rho* = 0.117  *p_raw_* = 0.478 | - |  |  |
| PFC Glx/tCr | *rho* = -0.095  *p_raw_* = 0.529 | *rho* = 0.019  *p_raw_* = 0.897 | - |  |
| SM1 Glx/tCr | *rho* = 0.115  *p_raw_* = 0.468 | *rho* = -0.238  *p_raw_* = 0.051 | *rho* = 0.062  *p_raw_* = 0.659 | - |

praw = uncorrected p value

Online Resource 8: correlations between grey matter (voxel fraction), neurochemical concentrations and inhibitory performance

|  | Age | GABA+/tCr | Glx/tCr | Flanker Effect  (ER) | Flanker Effect (RT) | Stroop Effect  (ER) | Stroop Effect (RT) | Go/no-go (ER) | Go/no-go (bis) |
| --- | --- | --- | --- | --- | --- | --- | --- | --- | --- |
| ***SM1 fGM*** |  |  |  |  |  |  |  |  |  |
| *Rho* | **-0.475** | 0.011 | 0.210 | -0.172 | -0.068 | 0.093 | **-0.255** | **-0.265** | -0.038 |
| *p* | **<0.001** | 0.931 | 0.079 | 0.150 | 0.575 | 0.440 | **0.032** | **0.027** | 0.757 |
| ***PFC fGM*** |  |  |  |  |  |  |  |  |  |
| *Rho* | -0.098 | 0.069 | 0.066 | -0.066 | -0.193 | 0.011 | 0.055 | -0.103 | -0.247 |
| *p* | 0.465 | 0.645 | 0.623 | 0.622 | 0.147 | 0.931 | 0.684 | 0.441 | 0.062 |

All measures were residual corrected for age (except age vs fGM comparisons, these are uncorrected bivariate correlations). Grey matter fraction (fGM); error rates (ER); reaction time (RT); balanced integrated score (bis).
